# Criminal Mania

**Published:** 1896-05-02

**Authors:** F. M. F. Skene


### Criminal Mania.

By F. M. F. Skene.

### IV.—ILLUSTRATIVE CASES.

Everyone would be disposed to endorse the verdict invariably given in cases of infanticide where children have been done to death by their own mother, that it has been the result of absolute, if even only temporary, insanity. It is a very natural conclusion. The instincts of motherhood are undeniably the strongest in human nature—women, as a rule, would give their own lives to protect their young, and when such powerful tendencies are suddenly transformed into impulses of murderous cruelty towards the helpless beings who have called forth their deepest affections, it would seem impossible to attribute such unnatural deeds to anything short of an irresistible criminal mania. Yet here is a case which proves that hatred and a passionate desire of revenge can prove mightier than all the tender instincts of maternity. A young woman was committed to prison, there to await her trial for the murder of her infant—her first-born child, only a few months old. She had nearly severed its head from its body, in the most cold-blooded and determined manner, and the judge and jury assembled to try her were unanimous in an immediate verdict of acquittal on the ground of insanity. Nevertheless, that woman was from first to last in full possession of her senses. Her act was premeditated for a deliberate purpose; it accomplished precisely the results she intended, and she never regretted it for a moment. Before she was removed to the lunatic asylum, where she was to lead a very comfortable existence, she told the prison visitor the whole truth, knowing well that such a confession could not injure her, and finding in it, apparently, a certain relief to her own mind. She was a handsome woman, with strongly-marked features, her straight black brows almost meeting over her sombre, dark eyes, and her whole expression indicating a most sullen and unforgiving temper. She had married a man many years older than herself, probably from motives of expediency, without having the smallest affection for him. Very soon this negative feeling passed into active hatred, for he was a man of hard nature, and apt to become very violent if she thwarted him. Quarrels were frequent between them before the birth of the child, but this event was a source, we believe, of great satisfaction to the husband. He would naturally be glad to have a son whom he could bring up to succeed him in his business, and he simply doted on the child, showing a much greater depth of affection than his wife seemed capable of feeling, and rousing in her a certain jealousy of the infant, whose claims upon him would evidently always be far superior to her own. At length there came a day when a most deadly quarrel took place between them, which left the woman with one deep concentrated desire for revenge dominating her whole being. She had soon made up her mind how best to attain her end. According to her own deliberate statement, she was too wholly given over to the longing for vengeance to feel either pity or tenderness as regarded the infant. With a refinement of malice she took one of her husband's razors with which to accomplish the ghastly deed. All this she related in a quiet, level tone of voice, and said frankly it had done all she wished, and she could not in the least regret it. If there was a God and a future state, as people said, the child was best off where it was, and she was free from her detested husband. She heard that this asylum was a very nice place, and she was quite content to go there. As to that hateful man, he was miserable enough, and always would be, she hoped most sincerely—and with that a grim smile passed over her face. The woman was perfectly sane; if ever anyone deserved to undergo the penalty of wilful murder she undoubtedly did, and her history shows the extreme difficulty of the diagnosis in judicial cases, which may or may not be the result of mental disease.

We can now give a typical instance of genuine criminal mania which sprang from that fruitful source of insanity—religious melancholy. A young man, whose education had been directed solely to the view of his adopting the clerical profession, had always been remarkable for his intense devotion to religion of the ultramontane type, and there came at length a time when he felt so overwhelmed with a sense of the wickedness of the world and the danger of falling personally into temptation, that he decided it would be best and safest for himself to die and end so difficult and dangerous a life; but he would not commit suicide, as he believed that he should then die in mortal sin, and so lose all hope of Paradise. He determined, therefore, to perpetrate some act which would ensure his incurring the punishment of death. A murder alone would effect this. His first idea was to kill a priest while ministering at the altar, so as to secure his instantaneous passage from the performance of the highest act of duty to its reward; but, on reflection, he remembered that the desecration of the sanctuary would be in itself a sin which he must avoid; he went, therefore, one night to the theatre, armed with a well-sharpened dagger, and looked round to make his selection; he saw a young girl, whose guileless expression seemed to denote her innocence and fitness for the mansions of the blest, while her exposed throat made her an easy prey to his knife. After rapidly muttering a prayer for a soul about to depart without warning, he plunged his weapon into her throat so scientifically that she fell dead on the spot without his having ever addressed a single word to her. When the true facts of the case were ascertained, the evidence of undoubted mania was too clear to be questioned for a moment, and the man was consigned to the asylum instead of the grave he had desired.

We could multiply instances that have occurred in real life, which show the extreme uncertainty of the diagnosis of criminal mania, and the great risk of attributing to mental disease that which is simply the result of human depravity.

